# Connected Speech Alterations and Progression in Patients With Primary Progressive Aphasia Variants

**DOI:** 10.1212/WNL.0000000000213524

**Published:** 2025-04-07

**Authors:** Elisa Canu, Federica Agosta, Laura Lumaca, Silvia Basaia, Veronica Castelnovo, Sofia Santicioli, Stefano Pisano, Elena Gatti, Alessandra Lamanuzzi, Edoardo Gioele Spinelli, Giordano Cecchetti, Francesca Caso, Giuseppe Magnani, Paola Caroppo, Sara Prioni, Cristina Villa, Stefano F. Cappa, Massimo Filippi

**Affiliations:** 1 Neuroimaging Research Unit, Division of Neuroscience, IRCCS San Raffaele Scientific Institute, Milan, Italy;; 2 Neurology Unit, IRCCS San Raffaele Scientific Institute, Milan, Italy;; 3 Vita-Salute San Raffaele University, Milan, Italy;; 4 Neurophysiology Service, IRCCS San Raffaele Scientific Institute, Milan, Italy;; 5 Unit of Neurology 5 - Neuropathology, Fondazione IRCCS Istituto Neurologico Carlo Besta, Milan, Italy;; 6 Department of Humanities and Life Sciences, University Institute for Advanced Studies IUSS Pavia, Italy;; 7 Dementia Research Center, IRCCS Mondino Foundation, Pavia, Italy; and; 8 Neurorehabilitation Unit, IRCCS San Raffaele Scientific Institute, Milan, Italy.

## Abstract

**Background and Objectives:**

Diagnosing the different variants of primary progressive aphasia (PPA) is challenging, but more accurate characterization can improve patient management and treatment outcomes. This study aimed to identify the following: (1) which speech features, alone or combined with language assessment and gray matter volumes (GMVs), best distinguish PPA variants and (2) how connected speech evolves in PPA.

**Methods:**

This prospective study was conducted at IRCCS San Raffaele Hospital in Milan, Italy, between 2010 and 2021. We included patients with PPA who underwent neuropsychological assessments, including standard evaluation of language and the “Picnic Scene” speech test, and, when available, brain structural MRI. Clinical and language assessments were also performed at follow-up in a subgroup. Sequential feature selection models identified speech parameters that best differentiated groups, incorporating age, sex, education, standard language tests, and GMVs. In each PPA group, linear mixed-effect models analyzed speech changes over time.

**Results:**

We included 95 patients with PPA (mean age 69 ± 9 years, 55 women [58%]; 40 with nonfluent variant PPA [nfvPPA], 35 with semantic variant PPA [svPPA], 20 with logopenic variant PPA [lvPPA]), of whom 82 underwent brain MRI and 34 had a follow-up visit after 10.2 months. Each model distinguished svPPA from the other PPA groups with high accuracy (*R*^2^ range 0.93–1.00; *p* < 0.001). No differences in accuracy were observed among models for this distinction. In differentiating nfvPPA and lvPPA groups, the models incorporating speech parameters (*R*^2^ = 0.92; *p* < 0.001), GMVs (*R*^2^ = 0.95; *p* < 0.001), and their combination (speech + GMVs; *R*^2^ = 0.97; *p* < 0.001) outperformed those using only standard language scores (*R*^2^ = 0.75; *p* = 0.01). Over time, patients with nfvPPA showed more phonological errors, the svPPA group exhibited more semantic and morphosyntactic errors along with difficulties in naming and syntax production, and patients with lvPPA exhibited reduced number of words per second and fewer words per sentence.

**Discussion:**

All models were equally effective in distinguishing the svPPA group from the other 2 PPA subtypes. However, compared with using standard measures alone, incorporating speech measures from the “Picnic Scene” speech test, GMVs, or their combination into the models significantly improved accuracy in differentiating nfvPPA and lvPPA groups. The PPA variants showed distinct speech trajectories. These variables can aid in understanding disease progression, predicting patient outcomes, and planning speech therapy interventions in clinical practice.

## Introduction

Primary progressive aphasia (PPA) is a neurodegenerative disorder characterized by the progressive and selective impairment of language production and/or comprehension. Current diagnostic criteria identify 3 distinct variants^1^: the nonfluent variant PPA (nfvPPA), characterized by agrammatism and/or apraxia of speech (AOS); the semantic variant PPA (svPPA), noted for anomia and difficulties in single-word comprehension; and the logopenic variant PPA (lvPPA), which presents with word-finding and sentence repetition difficulties. Pathologically, nfvPPA and svPPA are more likely to be associated with frontotemporal lobar degeneration (FTLD), including tau or TDP-43 pathology, while most cases of lvPPA are caused by Alzheimer disease.^2^ As disease-modifying drugs for these underlying pathologies begin to emerge,^3-5^ the need for accurate in vivo diagnosis of PPA phenotypes has become increasingly urgent.

Language assessment plays a crucial role in the clinical diagnosis and differentiation of the 3 PPA variants. Among various neuropsychological batteries, the analysis of extended speech production is particularly valuable. This analysis provides comprehensive information across different levels of language organization, including phonetic, phonological, lexicosemantic, and morphosyntactic aspects.^6^ Specifically, using a picture description paradigm, such as the Picnic Scene from the Western Aphasia Battery (WAB),^7^ facilitates the assessment of all these language levels.

Studies analyzing the speech of patients with PPA have shown that all PPA variants exhibit a reduced speech rate compared with healthy controls (HCs). Among the variants, patients with nfvPPA have the slowest speech rate, followed by those with lvPPA and svPPA.^8,9^ Compared with controls, nfvPPA cases are further characterized by frequent phonemic errors and a reduced ability to generate complex syntactic structures, such as the use of embeddings.^8,10-14^ By contrast, patients with svPPA use fewer nouns and more pronouns and have a higher mean frequency of produced words.^8,9,13,14^ Patients with lvPPA display an increased number of filled pauses, false starts, phonemic errors, and repaired sequences, similar to those with nfvPPA.^8,9,13,14^

The overlapping features in lvPPA and nfvPPA, such as slow speech production and reduced syntactic complexity, make distinguishing between these 2 variants particularly difficult.^6^ Among all speech characteristics, the most informative for differentiating lvPPA and nfvPPA is the presence of phonetic distortions, which are more frequent in the latter.^8^

However, although phonetic distortions are commonly observed in English-speaking patients with nfvPPA, they are rare in Italian cases,^15^ making differential diagnosis in our country still complex. Recent studies have significantly advanced clinical practice by improving the ability to characterize and differentiate these phenotypes in vivo. This has been achieved through a combination of standard and experimental language assessments, alongside single or multimodal neuroimaging techniques.^16-18^ Despite these substantial advancements, accurately distinguishing these clinical presentations remains challenging.

Furthermore, research on how speech evolves over time in patients with PPA is still limited.^19-21^ A deeper understanding of how language abilities change over time in these conditions could improve speech therapy interventions, enhance prognostic accuracy, and refine monitoring in treatment trials.

In this study, we aimed to identify which aspects of connected speech, whether used alone or in combination with standard language tests and/or brain gray matter volumes (GMVs), most effectively distinguish between the PPA variants. In addition, we conducted a longitudinal analysis on a subgroup of participants with PPA to investigate how connected speech evolves over time within each PPA variant.

## Methods

### Standard Protocol Approvals, Registrations, and Patient Consents

The ethical standards committee on human experimentation of IRCCS San Raffaele Scientific Institute (Milan, Italy) approved the study protocol, and all participants provided written informed consent before study inclusion.

### Participants

A total of 120 patients with a diagnosis of PPA^1^ were prospectively recruited from 2 referral clinics in Lombardy (Italy) and referred to IRCCS San Raffaele Hospital in Milan between 2010 and 2021 for a multimodal longitudinal prospective study, which involved neuropsychological and clinical assessments, and brain MRI. In both centers, the diagnosis of PPA variants was clinically reached according to the current criteria.^1^ It was supported by the available imaging (MRI and/or FDG-PET) and the CSF (details in the eMethods) profile when available and confirmed by a multidisciplinary team that included neurologists, neuropsychologists, speech pathologists, and neuroradiologists, all expert in neurodegeneration and PPA. From the large cohort, we included participants who had undergone a neurologic examination, a comprehensive neuropsychological assessment, and an audiorecorded “Picnic Scene” test from the WAB.^7^ Some patients also underwent a brain MRI scan, and, among them, some had a lumbar puncture as part of their diagnostic workup.^22^ Finally, a subgroup of patients underwent a neuropsychological follow-up visit, which included both standard tests and the “Picnic Scene” test.

The first group of HCs (HC-speech), who were similar to patients with PPA regarding sex, age, and education, was recruited among nonconsanguineous relatives and by word of mouth. They underwent a neuropsychological screening that included the Mini-Mental State Examination (MMSE), the Beck Depression Inventory (BDI), and the “Picnic Scene” test. A second group of HCs (HC-MRI) was retrospectively selected to match the PPA subgroup undergoing MRI scans regarding sex, age, education, and the MRI scanner used for data acquisition. Inclusion criteria for both HC groups were the following: age range 60–80 years; education range 5–18 years; MMSE score >27^23^; absence of mood disturbances as demonstrated by a score less than 15 in the BDI^24^; no family history of neurodegenerative diseases.

Participants (patients with PPA, HC-speech, and HC-MRI) were excluded if they had significant (other) medical illnesses or substance abuse that could interfere with cognitive functioning; any (other) major systemic, psychiatric, or neurologic illnesses; and (other) causes of focal or diffuse brain damage, including cerebrovascular disease at conventional MRI scans.

### Neuropsychological Assessment and Speech Evaluation

In all patients, the neuropsychological assessment investigated the global cognitive functioning, standard (confrontation naming, repetition, and single-word comprehension) and nonstandard language, verbal and nonverbal memory, attention and executive functions, visuospatial abilities, and behavior (full details are provided in the eMethods).

In patients with PPA and HCs, speech samples consisted of the oral description of the Picnic Scene of the WAB,^25^ which were audiorecorded, transcribed into an Excel file by expert speech therapists and neuropsychologists, and analyzed according to a previously described quantitative procedure.^8,15^ Four main domains were investigated: speech rate and speech sound errors; other disruptions to fluency, lexical content, and syntactic structure; and complexity. Full details are provided in the eMethods.

### MRI Acquisition and Analysis

Brain MRI scans of 82 patients with PPA and 61 HCs (HC-MRI group) were performed at IRCCS Scientific Institute San Raffaele, Milan, Italy. Using 2 different 3T Philips scanners (Ingenia CX, Intera), the following brain MRI sequences were obtained: 3D T1-weighted, 3D/2D fluid-attenuated inversion recovery, and 3D/2D T2-weighted sequences. Details on sequence parameters are provided in the eMethods.

### MRI Preprocessing

Single-subject 3D T1-weighted images were preprocessed as reported in eMethods.

#### Gray Matter Volumetry

In patients with PPA and the HC-MRI group, cortical GM maps were obtained from the segmentation step of SPM^26^ while the maps of the basal ganglia, hippocampus, and amygdala were obtained using the FMRIB's Integrated Registration and Segmentation Tool in FSL.^27^ The AAL atlas was then registered to individual T1-weighted images, masked using the GM maps, by means of linear (FLIRT)^28^ and nonlinear (FNIRT)^29^ registrations. GMVs were obtained and multiplied by the normalization factor derived from SIENAx to correct for head size.

#### Voxel-Based Morphometry

To provide the pattern of atrophy in each PPA group compared with the HC-MRI group, we performed voxel-based morphometry as detailed in the eMethods.

### Statistical Analysis: Cross-Sectional

#### Sociodemographic, Clinical, and Cognitive Data Analysis

Sociodemographic and clinical data were compared among groups (HC-speech, nfvPPA, svPPA, lvPPA) using analysis of variance (ANOVA) models. The Fisher exact test was used for categorical variables. Neuropsychological differences were assessed using analysis of covariance (ANCOVA) models accounting for age, sex, and education, followed by post hoc pairwise comparisons (Bonferroni-corrected for multiple comparisons [number of groups], *p* < 0.05).

#### Variable Preparation for the Sequential Feature Selection Models

All variables were prepared and then selected for the sequential feature selection (SFS) analysis. To this purpose, the following procedure was applied: first, we conducted an outlier evaluation; second, we identified the variables from the “Picnic Scene” test and the GMVs that exhibited significant differences between patients and HCs and/or among patient groups; third, we assessed multicollinearity (among variables identified at the second step) in the HC groups using Pearson correlation analysis. Details are provided in the eMethods.

#### SFS Models

A SFS, using a logistic regression model, was applied to differentiate each PPA variant from the HC-speech group and from each other. This is a model-based feature selection method that selects a subset of features that are most predictive of the outcome while reducing the complexity of the model. During the SFS process, we used ridge regression (L2 penalty, regularization strength parameter = 1), which penalizes large coefficients without completely excluding features. Specifically, we applied a forward SFS model that iteratively adds features to the model, selecting those that most improve performance. In each model, 2 groups at once were considered as the dependent variables and candidate independent variables included sociodemographic features (sex, age, education), standard language scores (naming, repetition, and single-word comprehension), speech measures, and GMVs. Separate models were run for each independent variable alone (speech, standard scores, and GMVs) and for any combination of them. The candidate independent variables were selected among those found significantly different among groups at the baseline, excluding those multicollinear. Models considering standard language scores and/or GMVs were not run when comparisons with HC-speech were performed. An *R*^2^ goodness-of-fit statistic was estimated for each model. For each model, 10-fold cross-validation was performed. This means that the data set was portioned into 10 equally sized folds, iteratively training the model on 9 folds and validating it on the remaining fold. This process was repeated 10 times, with each fold serving as the validation set exactly once. By averaging the performance metrics across all folds, the risk of overfitting the model was reduced. It is important to note that the number of variables entered into each model was dynamically determined by the SFS process and was manually limited to a maximum of 6 features to ensure the model's robustness. A 2-sided *p* value <0.05 was considered statistically significant. Analyses were performed using Python (version 3.10, library mlxtend). To compare the predictive accuracy of each of the 2 pairs of models, which were run separately for each dependent variable and for any combination of them, the McNemar test was applied. The McNemar test is based on a 2 × 2 contingency table of the 2 models' predictions. The significance level for all the statistical tests used was set at *p* < 0.05.

### Statistical Analysis: Longitudinal

Baseline sociodemographic and clinical data of the patients who underwent the follow-up visit (10 patients with nfvPPA, 17 with svPPA, 7 with lvPPA) were compared with the HC-speech group using 1-way ANOVA models. The Fisher exact test was used for the analysis of categorical variables. Between-group differences in the baseline performances at the Picnic Scene test and at the neuropsychological evaluation were assessed using ANCOVA models, followed by post hoc pairwise comparisons (Bonferroni-corrected for multiple comparisons [number of groups], *p* < 0.05) accounting for age, sex, and education.

Longitudinal changes on speech parameters and standard language performances over time were investigated in all PPA variants together and in each PPA group using the test for linear trend. Group-by-time interaction was also performed to evaluate longitudinal between-group differences. In these analyses, age, sex, education, and time between visits were included as covariates. Random effect of subject (ID) for each model has been considered. *p* Values were Bonferroni-corrected for multiple comparisons (number of groups) at < 0.05. All statistical analyses were performed using R Statistical Software (version 4.0.3; R Foundation for Statistical Computing, Vienna, Austria).

### Data Availability

Anonymized data not published within this article will be made available by request from any qualified investigator.

## Results

### Baseline Clinical Findings

#### Sample

Based on inclusion criteria, 95 patients (40 patients with nfvPPA [age 70 ± 9, 24 women, Clinical Dementia Rating (CDR)-FTLD 4 ± 3], 35 with svPPA [age 67 ± 9, 20 women, CDR-FTLD 6 ± 5], and 20 with lvPPA [age 72 ± 7, 11 women, CDR-FTLD 4 ± 2]) were involved in the study. Eighty-two patients (37 with nfvPPA, 30 with svPPA, and 15 with lvPPA) also underwent a brain MRI scan, and 47 patients of them (22 with nfvPPA, 12 with svPPA, and 13 with lvPPA) underwent a lumbar puncture.^22^ Concerning HCs, we recruited 23 HC-speech participants and we retrospectively selected 61 HC-MRI participants. Details on the sample have also been provided in the eMethods.

Sociodemographic and clinical characteristics of patients with PPA and the HC-speech group are presented in Table 1. Patients and controls were matched for age, sex, and education. Compared with the HC-speech group, the 3 groups of patients performed significantly worse at the MMSE. No HC-speech participant presented depressive symptoms assessed with BDI. PPA groups were comparable among each other regarding age, sex, education, and disease severity assessed with MMSE, CDR, CDR-Sum of Boxes, and CDR-FTD. Patients with svPPA had longer disease duration compared with the nfvPPA group and less autonomy in the IADL compared with patients with lvPPA. Compared with the other variant groups, patients with lvPPA showed a higher ratio of CSF levels of phosphorylated tau to β-amyloid 42.

**Table 1:** Sociodemographic and Clinical Features of the Sample at Baseline

s	HC-speech (N = 23)	nfvPPA (N = 40)	svPPA (N = 35)	lvPPA (N = 20)	*p* Value					
					nfvPPA vs HC	svPPA vs HC	lvPPA vs HC	nfvPPA vs svPPA	nfvPPA vs lvPPA	svPPA vs lvPPA
Age (y)	66.2 ± 6.3 (56.5–81.4)	70.2 ± 8.5 (51.6–3.9)	66.6 ± 8.7 (42.1–81.6)	72.4 ± 7.0 (56.4–81.3)	0.34	1.00	0.07	0.31	1.00	0.06
Sex (female/male)	13/10	24/16	20/15	11/9	0.80	1.00	1.00	0.82	0.79	1.00
Education (y)	9.5 ± 3.3 (5.0–17.0)	9.7 ± 4.9 (3.0–22.0)	11.9 ± 4.6 (5.0–18.0)	12.1 ± 3.6 (5.0–17.0)	1.00	0.24	0.36	0.17	0.31	1.00
Disease duration (mo)	—	28.91 ± 14.10 (3.0–68.0)	45.92 ± 23.31 (11.3–126.6)	33.84 ± 19.74 (4.1–62.1)	—	—	—	0.002	1.00	0.12
CDR	—	0.4 ± 0.5 (0.0–2.0)	0.8 ± 0.7 (0.0–2.0)	0.5 ± 0.3 (0.0–1.0)	—	—	—	0.09	1.00	0.53
CDR-SB	—	2.0 ± 1.8 (0.0–8.5)	3.5 ± 3.5 (0.5–10.5)	2.2 ± 1.3 (0.50–4.0)	—	—	—	0.17	1.00	0.53
CDR-FTLD	—	4.0 ± 2.9 (0.5–11.5)	5.6 ± 4.7 (1.0–14.5)	4.0 ± 2.0 (2.0–6.0)	—	—	—	0.89	1.00	1.00
CSF (p-tau/Aβ42, pg/mL)^a^	—	0.08 ± 0.04 (0.04–0.19)	0.06 ± 0.04 (0.04–0.17)	0.23 ± 0.12 (0.07–0.46)	—	—	—	1.00	<0.001	0.001
MMSE score	29.3 ± 0.1 (27.0–30.0)	23.7 ± 5.8 (5.0–30.0)	20.9 ± 7.8 (5.0–30.0)	22.7 ± 5.4 (14.0–29.0)	0.003	<0.001	0.004	0.35	1.00	1.00
ADL	—	5.8 ± 0.5 (4.0–6.0)	5.6 ± 1.0 (2.0–6.0)	6.0 ± 0.0 (6.0–6.0)	—	—	—	0.59	1.00	0.27
IADL^b^	—	85.9 ± 26.7 (0.0–100.0)	74.8 ± 32.0 (0.0–100.0)	99.1 ± 3.3 (87.5–100.0)	—	—	—	0.33	0.38	0.02
BDI score	7.9 ± 4.2 (0.0–14.0)	—	—	—	—	—	—	—	—	—

Abbreviations: Aβ42 = amyloid β42; ADL = activities of daily living; ANOVA = analysis of variance; BDI = Beck Depression Inventory; CDR = Clinical Dementia Rating; CDR-FTLD = Clinical Dementia Rating–Frontotemporal Lobar Degeneration; CDR-SB = Clinical Dementia Rating Sum of Boxes; HC = healthy control; IADL = instrumental activities of daily living; lvPPA = logopenic variant of primary progressive aphasia; MMSE = Mini-Mental State Examination; nfvPPA = nonfluent/agrammatic variant of primary progressive aphasia; p-tau = phosphorylated tau; svPPA = semantic variant of primary progressive aphasia.

Values are numbers or mean ± SD (range). Disease duration was defined as months from onset to the date of neuropsychological visit. We considered significant *p* values less than 0.05. *p* Values (*p* < 0.05) refer to ANOVA models followed by post hoc pairwise comparisons (Bonferroni-corrected for multiple comparisons) or the Fisher exact test.

a Forty-seven patients (22 with nfvPPA, 12 with svPPA, and 13 with lvPPA) underwent lumbar puncture.

b Ratio between the score obtained by the patient and the maximum number of administered items × 100.

The 3 groups of patients with PPA performed similarly in almost all considered neuropsychological tests, except for a worse performance of patients with nfvPPA in the digit span forward compared with patients with svPPA and a worse performance of patients with svPPA in confrontation naming, single-word comprehension, semantic fluency, and semantic knowledge, compared with patients with other PPA variants (eTable 1).

#### Speech Evaluation

The extracted speech features from the “Picnic Scene” test are provided in Table 2. The performance of patients with PPA and HC-speech participants on the “Picnic Scene” test is presented in Table 3. Compared with the HC-speech group, all patients with PPA variants showed significant differences in most speech parameters.

**Table 2:** Speech Parameters Obtained From the Picnic Scene Test

Variable	Abbreviation	Definition	Formula
Speech rate and speech sound errors			
Total duration (s)	—	Duration of the sample in seconds of the participant's production, excluding moments when the participant does not attempt to speak, interventions by the experimenter, comments directed at the examiner (e.g., “Should I continue?”), or remarks about their own difficulties (e.g., “I can't find the words”)	—
Duration without pauses (s)	—	The total duration of the sample excluding pauses (whether filled or unfilled)	Total duration of the sample pauses
Total number of words	—	The number of words identified based on orthographic boundaries	—
Words per second	WPS		Total number of words/total duration
Words per second-no pauses	WPS-np		Total number of words/duration without pauses
Phonotactic distortions	—	The number of distortions produced, defined as the production of a phone or group of phones that do not belong to the phonetic inventory of the Italian language	—
Apraxia of speech	AOS		Distortions/total number of words × 100
Phonological errors	PE	Number of words containing omissions, repetitions, transpositions, or substitutions of phonemes and neologisms. Words containing multiple phonological errors are counted only once	—
Phonological errors per word	PEW		Phonological errors/total number of words
Other disruptions to fluency			
False starts	—	Number of words pronounced partially. These are not included in the total word count	
False start rate	—		False starts/total duration
Filled pauses	—	Number of nonmeaningful sounds (such as “um,” “mm,” and “eh”)	
Filled pause rate	—		Filled pauses/total duration
Self-corrected sequences	SCS	Attempts in sequence of 1 or more complete words that result in redundancy or incorrectness	
SCS rate	SCS rate		Self-corrected sequences/total duration
Incomplete sentences	IS	Sentences abandoned after the subject and verb have been produced	
Incomplete sentence rate	IS rate		Incomplete sentences/total duration
Lexical content			
Open class words	—	Nouns, verbs (excluding the auxiliary verbs “to be” and “to have”), qualifying adjectives, and derived adverbs	
Closed class words	—	Propositions, conjunctions, articles, pronouns, possessive adjectives, numerals, demonstratives, interrogatives, indefinite pronouns, and the verbs “to be” and “to have” both when functioning as auxiliaries and when serving as main verbs in the sentence	
Verbs	—	Number of verbs produced (verbs such as “is playing” are counted as a single verb)	
Verb rate	—		Verbs/total duration
Nouns	—	Number of nouns produced	
Noun rate	—		Nouns/total duration
Open class word ratio	rOC		Open class words/closed class words
Closed class word ratio	rCC		Closed class words/open class words
Verb proportion	PrV		Verbs/(verbs + nouns)
Mean frequency of nouns	—	Frequency of use of Italian lemmas	
Logarithmic frequency of nouns	Log frequency		Log_10_ (mean frequency of nouns)
Semantic errors	SemE	Errors in which a word is replaced by another with a different semantic significance or an improper semantic choice	
SemE per word	SemEW		SemE/total number of words
Syntactic structure and complexity			
Utterances	—	Sequence of words uninterrupted by unnatural pauses, such as prosodic and intonational changes, or other pauses. Each utterance is reported in a separate row in the transcription sheet.	
Utterance length	UL		Total number of words/utterances
Sentences	—	Syntactic structures consisting of a subject and a predicate	
Sentence ratio	—		Sentences/total duration
Number of words in sentences	NWS	Number of words in each sentence, excluding the words that form self-corrected sequences	
Mean length of sentences	SL		Number of words in sentences/sentences
Sentence ratio	rSentences		Sentences/utterances
Embeddings	—	Sentences that depend on a main sentence to complete their meaning	
Sentences with express subject	SES	Sentence in which the subject is phonetically realized by a noun or a personal pronoun	
Sentences with implied subject	SIS	Clauses in which the subject has no phonetic realization (this can occur in Italian language)	
Proportion of sentences with expressed subject	PrSES		SES/(SES + SIS)
Syntax production rate	SP rate		Number of words in sentences/number of words
Morphosyntactic errors	MsynE	Omission of articles and prepositions, errors in verb inflection or gender and number agreement or alteration of word order	
Morphosyntactic error ratio	rMsynE		Morphosyntactic errors/number of words in sentences

**Table 3:** Performance of Patients With PPA and HC-Speech Participants on the “Picnic Scene” Test

	HC-speech (N = 23)	nfvPPA (N = 40)	svPPA (N = 35)	lvPPA (N = 20)	*p* Value					
					nfvPPA vs HC	svPPA vs HC	lvPPA vs HC	nfvPPA vs svPPA	nfvPPA vs lvPPA	svPPA vs lvPPA
Speech rate and speech sound errors										
Total duration (s)	74.35 ± 18.93 (37.00–108.00)	116.41 ± 45.99 (38.00–272.00)	117.63 ± 49.22 (40.00–270.00)	158.35 ± 78.66 (50.00–331.00)	0.02	0.02	<0.001	1.00	0.03	0.05
Duration without pauses (s)	55.78 ± 20.15 (15.00–87.00)	55.83 ± 28.58 (17.00–133.00)	65.91 ± 29.52 (19.00–123.00)	101.80 ± 58.20 (29.00–223.00)	1.00	1.00	0.003	1.00	<0.001	0.01
Total number of words	126.87 ± 42.54 (40.00–208.00)	78.4 ± 36.58 (18.00–186.00)	138.89 ± 66.41 (40.00–267.00)	154.7 ± 77.85 (41.00–339.00)	0.01	1.00	1.00	<0.001	<0.001	1.00
WPS	1.70 ± 0.39 (0.9–2.32)	0.75 ± 0.35 (0.1–1.6)	1.25 ± 0.49 (0.48–2.35)	1.03 ± 0.34 (0.24–1.76)	<0.001	<0.001	<0.001	<0.001	0.18	0.44
WPS-np	2.33 ± 0.35 (1.63–2.96)	1.47 ± 0.41 (0.45–2.44)	2.11 ± 0.40 (1.16–2.90)	1.59 ± 0.28 (1.08–2.26)	<0.001	0.25	<0.001	<0.001	1.00	<0.001
Phonotactic distortions	0 ± 0 (0.00–0.00)	0.3 ± 1.04 (0.00–6.00)	0.06 ± 0.24 (0.00–1.00)	0.15 ± 0.37 (0.00–1.00)	0.44	1.00	1.00	1.00	1.00	1.00
AOS	0.00 ± 0.00 (0.00–0.00)	0.48 ± 1.6 (0.00–7.41)	0.07 ± 0.27 (0.00–1.2)	0.12 ± 0.29 (0.00–0.92)	0.31	1.00	1.00	0.64	1.00	1.00
PE	0.39 ± 0.94 (0.00–4.00)	5.35 ± 7.96 (0.00–33.00)	0.71 ± 1.02 (0.00–4.00)	3.2 ± 3.82 (0.00–16.00)	0.002	1.00	0.40	0.002	1	0.57
PEW	0.00 ± 0.01 (0.00–0.04)	0.09 ± 0.16 (0.00–0.71)	0.01 ± 0.01 (0.00–0.05)	0.03 ± 0.03 (0.00–0.15)	0.01	1.00	1.00	0.01	0.21	1.00
Other disruptions to fluency										
False starts	1.00 ± 1.00 (0.00–3.00)	4.83 ± 7.09 (0.00–36.00)	2.63 ± 3.77 (0.00–19.00)	7.45 ± 5.38 (1.00–23.00)	0.05	1.00	0.002	0.53	0.53	0.02
False start rate	0.01 ± 0.01 (0.00–0.03)	0.05 ± 0.08 (0.00–0.35)	0.02 ± 0.02 (0.00–0.12)	0.05 ± 0.03 (0.01–0.10)	0.05	1.00	0.21	0.12	1.00	0.37
Filled pauses	11.7 ± 6.11 (3.00–26.00)	12.68 ± 12.80 (0.00–54.00)	10.11 ± 6.03 (2.00–30.00)	21.35 ± 13.46 (0.00–45.00)	1.00	1.00	0.045	1.00	0.04	0.002
SCS	5.35 ± 3.08 (0.00–12.00)	8.68 ± 8.14 (0.00–35.00)	10.66 ± 9.76 (1.00–45.00)	15.65 ± 9.46 (2.00–35.00)	1.00	0.25	0.005	1.00	0.06	0.37
SCS rate	0.07 ± 0.04 (0.00–0.16)	0.09 ± 0.12 (0.00–0.60)	0.09 ± 0.07 (0.01–0.37)	0.11 ± 0.06 (0.03–0.24)	1.00	1.00	1.00	1.00	1.00	1.00
IS	1.48 ± 1.34 (0.00–4.00)	0.73 ± 1.20 (0.00–4.00)	3.23 ± 2.78 (0.00–13.00)	2.5 ± 1.82 (0.00–7.00)	0.65	0.01	1.00	<0.001	0.02	0.75
IS rate	0.02 ± 0.02 (0.00–0.05)	0.01 ± 0.02 (0.00–0.08)	0.03 ± 0.03 (0.00–0.11)	0.02 ± 0.02 (0.00–0.06)	0.12	0.80	1.00	<0.001	0.56	0.31
Lexical content										
Open class words	47.17 ± 16.28 (18.00–85.00)	28.05 ± 11.64 (7.00–64.00)	36.74 ± 18.08 (15.00–92.00)	50.3 ± 27.36 (18.00–115.00)	<0.001	0.07	1.00	0.62	0.001	0.07
Closed class words	79.52 ± 27.16 (22.00–124.00)	48.65 ± 28.25 (2.00–129.00)	101.03 ± 50.83 (24.00–203.00)	103.75 ± 51.46 (22.00–223.00)	0.03	0.60	0.71	<0.001	<0.001	1.00
Verbs	21.26 ± 7.01 (5.00–32.00)	12.18 ± 8.17 (0.00–38.00)	25.6 ± 13.12 (8.00–55.00)	25.6 ± 11.84 (5.00–47.00)	0.01	0.90	1.00	<0.001	<0.001	1.00
Nouns	32.43 ± 10.97 (14.00–52.00)	20.95 ± 10.48 (1.00–56.00)	22.31 ± 11.38 (4.00–49.00)	34.05 ± 16.66 (11.00–75.00)	<0.001	0.006	1.00	1.00	0.004	0.01
Noun rate	0.44 ± 0.10 (0.26–0.60)	0.19 ± 0.09 (0.02–0.38)	0.21 ± 0.11 (0.06–0.48)	0.23 ± 0.07 (0.07–0.36)	<0.001	<0.001	<0.001	1.00	1.00	1.00
rOC	0.62 ± 0.14 (0.42–1.00)	0.91 ± 1.27 (0.14–8.00)	0.39 ± 0.12 (0.19–0.71)	0.50 ± 0.13 (0.29–0.82)	0.76	0.80	1.00	0.005	0.17	1.00
rCC	1.69 ± 0.33 (1.00–2.39)	1.80 ± 1.13 (0.13–7.14)	2.82 ± 0.97 (1.41–5.36)	2.13 ± 0.57 (1.22–3.4)	1.00	<0.001	0.56	<0.001	0.85	0.06
PrV	0.40 ± 0.07 (0.26–0.53)	0.37 ± 0.18 (0.00–0.83)	0.54 ± 0.13 (0.3–0.83)	0.43 ± 0.08 (0.23–0.62)	1.00	<0.001	1.00	<0.001	0.44	0.03
Mean frequency	83.54 ± 17.75 (51.15–123.3)	94.80 ± 75.01 (18.83–446.60)	242.35 ± 161.82 (28.33–627.00)	135.75 ± 119.20 (57.24–608.00)	1.00	<0.001	0.21	<0.001	0.39	0.01
Log frequency	1.91 ± 0.09 (1.71–2.09)	1.89 ± 0.27 (1.27–2.65)	2.28 ± 0.33 (1.45–2.80)	2.05 ± 0.23 (1.76–2.78)	1.00	<0.001	0.08	<0.001	0.03	0.04
SemE	0.91 ± 1.28 (0.00–4.00)	2.3 ± 2.16 (0.00–10.00)	1.77 ± 2.35 (0.00–12.00)	1.45 ± 1.73 (0.00–7.00)	0.04	0.22	1.00	1.00	1.00	1.00
SemEW	1.1 ± 0.01 (0.00–0.03)	0.03 ± 0.04 (0.00–0.22)	0.01 ± 0.01 (0.00–0.05)	0.01 ± 0.01 (0.00–0.04)	<0.001	1.00	1.00	0.002	0.004	1.00
Syntactic structure and complexity										
Utterances	34.43 ± 10.84 (14.00–60.00)	28.55 ± 10.30 (14.00–56.00)	35.34 ± 15.70 (12.00–79.00)	45.95 ± 19.80 (14.00–95.00)	0.57	1.00	0.23	0.58	0.001	0.08
UL	3.72 ± 0.77 (2.38–5.15)	2.77 ± 1.04 (0.97–5.22)	3.80 ± 0.98 (1.68–6.00)	3.32 ± 0.80 (1.78–4.60)	0.002	1.00	1.00	<0.001	0.16	0.54
Sentences	15.04 ± 5.18 (6.00–28.00)	9.25 ± 6.08 (0.00–23.00)	15.51 ± 7.04 (6.00–37.00)	15.80 ± 5.92 (5.00–26.00)	0.006	1.00	1.00	<0.001	0.001	1.00
NWS	108.43 ± 39.30 (39.00–199.00)	46.43 ± 37.62 (0.00–171.00)	99.11 ± 54.31 (31.00–216.00)	106.2 ± 56.65 (13.00–213.00)	<0.001	1.00	1.00	<0.001	<0.001	1.00
SL	7.81 ± 3.38 (3.5–16.11)	4.47 ± 1.96 (0.00–9.00)	6.21 ± 1.52 (3.64–9.71)	6.61 ± 3.44 (2.60–19.36)	<0.001	0.02	0.16	0.07	0.07	1.00
rSentences	0.46 ± 0.16 (0.24–0.78)	0.34 ± 0.22 (0.00–0.79)	0.46 ± 0.14 (0.25–0.92)	0.37 ± 0.13 (0.22–0.71)	0.048	1.00	1.00	0.002	1.00	0.50
Embeddings	7.09 ± 4.4 (1.00–16.00)	1.85 ± 2.33 (0.00–8.00)	6.43 ± 4.99 (0.00–21.00)	6.1 ± 5.33 (0.00–19.00)	<0.001	1.00	1.00	<0.001	0.007	1.00
SES	10.04 ± 4.62 (1.00–19.00)	7.05 ± 5.76 (0.00–21.00)	10.91 ± 7.12 (0.00–35.00)	12.6 ± 6.11 (1.00–21.00)	0.69	1.00	0.45	0.07	0.003	1.00
SIS	4.96 ± 3.43 (0.00–15.00)	1.88 ± 1.56 (0.00–6.00)	3.29 ± 2.24 (0.00–9.00)	2.35 ± 2.48 (0.00–7.00)	<0.001	0.09	0.008	0.07	1.00	1.00
PrSES	0.68 ± 0.22 (0.06–1.00)	0.61 ± 0.38 (0.00–1.00)	0.72 ± 0.23 (0.00–1.00)	0.82 ± 0.17 (0.50–1.00)	1.00	1.00	0.22	0.75	0.02	0.61
SP rate	0.86 ± 0.08 (0.67–0.99)	0.50 ± 0.30 (0.00–0.92)	0.71 ± 0.14 (0.32–0.91)	0.67 ± 0.13 (0.32–0.93)	<0.001	0.10	0.06	<0.001	0.02	1.00
MsynE	1.7 ± 1.96 (0.00–6.00)	3.58 ± 3.54 (0.00–16.00)	3.83 ± 2.57 (0.00–10.00)	4.2 ± 2.75 (0.00–9.00)	0.10	0.04	0.04	1.00	1.00	1.00
rMsynE	0.01 ± 0.03 (0.00–0.10)	0.25 ± 0.65 (0.00–4.00)	0.04 ± 0.06 (0.00–0.20)	0.06 ± 0.11 (0.00–0.50)	0.13	1.00	1.00	0.30	0.89	1.00

Abbreviations: ANCOVA = analysis of covariance; AOS = apraxia of speech; HC = healthy control; IS = incomplete sentences; log frequency = frequency logarithm; lvPPA = logopenic variant of PPA; MsynE = morphosyntactic errors; nfvPPA = nonfluent/agrammatic variant of primary progressive aphasia; NWS = number of words in sentences; PE = phonological errors; PEW = phonological errors per word; PPA = primary progressive aphasia; PrSES = proportion of sentences with expressed subject; PrV = proportion of verbs; rCC = closed class word ratio; rMsynE = morphosyntactic error ratio; rOC = open class word ratio; rSentences = sentence ratio; SCS = self-corrected sequences; SemE = semantic errors; SemEW = semantic errors per word; SES = sentences with express subject; SIS = sentences with implied subject; SL = sentence length; SP rate = syntax production rate; svPPA = semantic variant of PPA; UL = utterance length; WPS = words per second; WPS = words per second-no pauses.

Values are mean ± SD (range). We considered significant *p* values less than 0.05. *p* Values (*p* < 0.05) refer to ANCOVA models followed by post hoc pairwise comparisons (Bonferroni-corrected for multiple comparisons) accounted for age, sex, and education.

Specifically, compared with the HC-speech group, patients with nfvPPA pronounced fewer words (total number of words, words in sentences, open and closed class words, nouns, verbs) in more time (higher total duration of speech) with altered production rate (fewer words per second [WPS], fewer words per seconds in the speech sample with no pauses [WPS-np], and reduced syntax production rate). Patients with nfvPPA also produced more phonological errors, shorter utterances, fewer and shorter sentences (embeddings, sentences with implied subject), and more semantic errors. Compared with the HC-speech group, patients with svPPA showed a higher total duration of speech and produced fewer WPS, more incomplete sentences and fewer nouns, an increased closed class word ratio and verbs, a higher mean frequency (and frequency logarithm) of the pronounced nouns, shorter sentence length, and more morphosyntactic errors. Finally, compared with the HC-speech group, patients with lvPPA showed a higher total duration of speech with and without pauses and produced fewer WPS and WPS-np, more false starts, filled pauses, self-corrected sequences, morphosyntactic errors, and fewer sentences with implied subject.

Considering the comparisons among the 3 PPA variants, compared with lvPPA and svPPA groups, patients with nfvPPA produced fewer words (total number of words), more incomplete sentences, fewer closed class words and verbs, fewer sentences and words in sentences, fewer embeddings, fewer sentences with express subject, lower frequency logarithm of the pronounced nouns, and a reduced syntax production rate. In addition, compared with the svPPA group, patients with nfvPPA produced more phonological errors per words and showed reduced incomplete sentence rate, number of verbs, and sentence rate. Compared with nfvPPA and svPPA groups, patients with lvPPA showed a longer duration of speech without pauses and they produced more filled pauses and nouns; the logarithmic frequency was higher than that of patients with nfvPPA and lower than that of those with svPPA. Compared with lvPPA and nfvPPA groups, patients with svPPA showed higher WPS-np, higher mean frequency (and frequency logarithm) of pronounced nouns, and a higher proportion of verbs.

### MRI Findings

#### Voxel-Based Morphometry

The patterns of GM atrophy observed in each PPA group (n = 82) when compared with HC-MRI participants were consistent with those already reported^1^ (eFigure 1).

#### Gray Matter Volumetric Measures

eTable 2 reports the regions that were found to be significantly different in at least 1 comparison between groups. Compared with the HC-MRI group, each group of PPA showed a widespread pattern of reduced volumetry, mainly in the left hemisphere. Furthermore, compared with lvPPA and svPPA groups, patients with nfvPPA showed reduced volumetry of the bilateral pars opercularis of the inferior frontal gyrus and of the left supplementary motor area. Compared with nfvPPA and lvPPA groups, patients with svPPA exhibited a more pronounced atrophy in the left insula, left middle and inferior temporal gyrus, bilateral hippocampus, bilateral parahippocampal and fusiform gyri, bilateral amygdala, and bilateral superior and middle temporal poles. Patients with lvPPA displayed a pattern of atrophy that was more prominent in the left middle temporal and occipital gyri compared with patients with nfvPPA. Finally, compared with nfvPPA and svPPA groups, patients with lvPPA showed greater volume of the left putamen.

### Multicollinearity of Data

Highly correlated variables and final selected variables for the main SFS models are reported in eTable 3. Details are provided in the eMethods.

### SFS Findings

The prediction models resulting from the SFS are summarized in Table 4.

**Table 4:** Sequential Feature Selection Models for Group Distinction Using “Picnic Scene” (Speech) Parameters, Standard Language Scores, and/or Gray Matter Volumes as Dependent Variables

Model	*R* ^2^	*p* Value	Variables
HC-speech vs nfvPPA			
Speech	0.97	<0.001	Education, WPS-np, verbs, SL
HC-speech vs svPPA			
Speech	0.93	<0.001	Sex, total duration, rOC, total number of words, mean frequency
HC-speech vs lvPPA			
Speech	0.98	<0.001	WPS-np, false starts, mean frequency
nfvPPA vs svPPA			
Speech	0.94	<0.001	WPS-np, PrV, IS, mean frequency
Speech + standard language	0.96	<0.001	Age, total duration, WPS, WPS-np, repetition, naming
Speech + GMVs	0.99	<0.001	Sex, IS, left middle frontal gyrus, left middle temporal pole
Speech + standard language + GMVs	0.99	<0.001	Sex, IS, left middle frontal, left middle temporal pole
Standard language	0.94	<0.001	Repetition, naming
GMVs	0.97	<0.001	Left middle frontal, left middle temporal pole
GMVs + standard language	0.97	<0.001	Left middle frontal, left middle temporal pole
nfvPPA vs lvPPA			
Speech	0.92	<0.001^a^	Sex, total duration, rOC, utterances
Speech + standard language	0.92	<0.001^a^	Sex, total duration, rOC, utterances
Speech + GMVs	0.97	<0.001^a^	Sex, utterances, sentences, L SMA, L insula, L middle temporal
Speech + standard language + GMVs	0.97	<0.001^a^	Sex, utterances, sentences, L SMA, L insula, L middle temporal
Standard language	0.75	0.01	Sex, repetition, naming, single-word comprehension
GMVs	0.95	<0.001^a^	Education, R pars opercularis, L insula, L hippocampus, L inferior temporal, R inferior temporal
GMVs + standard language	0.95	<0.001	Education, R pars opercularis, L insula, L hippocampus, L inferior temporal, R inferior temporal
lvPPA vs svPPA			
Speech	0.96	<0.001	Age, PE, false starts, filled pauses, rSentences, MsynE
Speech + standard language	0.93	<0.001	WPS-np, IS, SP rate, naming
Speech + GMVs	1.00	<0.001	Age, WPS, WPS-np, UL, SES, left middle temporal pole
Speech + standard language + GMVs	1.00	<0.001	Age, WPS, WPS-np, UL, SES, left middle temporal pole
Standard language	0.90	<0.001	Repetition, naming
GMVs	1.00	<0.001	Sex, education, L insula, L superior temporal, L middle temporal pole, R middle temporal pole
GMVs + standard language	1.00	<0.001	Sex, education, L insula, L superior temporal, L middle temporal pole, R middle temporal pole

Abbreviations: GMVs = gray matter volumes; IS = incomplete sentences; L = left; lvPPA = logopenic variant of PPA; MsynE = morphosyntactic errors; nfvPPA = nonfluent/agrammatic variant of PPA; PE = phonological errors; PPA = primary progressive aphasia; PrV = proportion of verbs; R = right; rOC = open class words ratio; rSentences = sentence ratio; SES = sentences with express subject; SP rate = syntax production rate; svPPA = semantic variant of PPA; Temporal Mid = middle temporal gyrus; UL = utterance length; WPS = words per second; WPS = words per second-no pauses.

Sociodemographic features were consistently included in all models. *R*^2^ reflects the accuracy of the model.

a Significant differences (McNemar *p* < 0.05) compared with the model including standard language scores only.

The best model differentiating patients with nfvPPA from HC-speech participants included education, WPS-np, verbs, and sentence length (*R*^2^ = 0.97; *p* < 0.001). The best model differentiating patients with svPPA from HC-speech participants included sex, total duration of speech, open class word ratio, total number of words, and mean frequency (*R*^2^ = 0.93; *p* < 0.001). The best model differentiating patients with lvPPA from HC-speech participants included WPS-np, false starts, and mean frequency (*R*^2^ = 0.98; *p* < 0.001).

**Figure 1:**
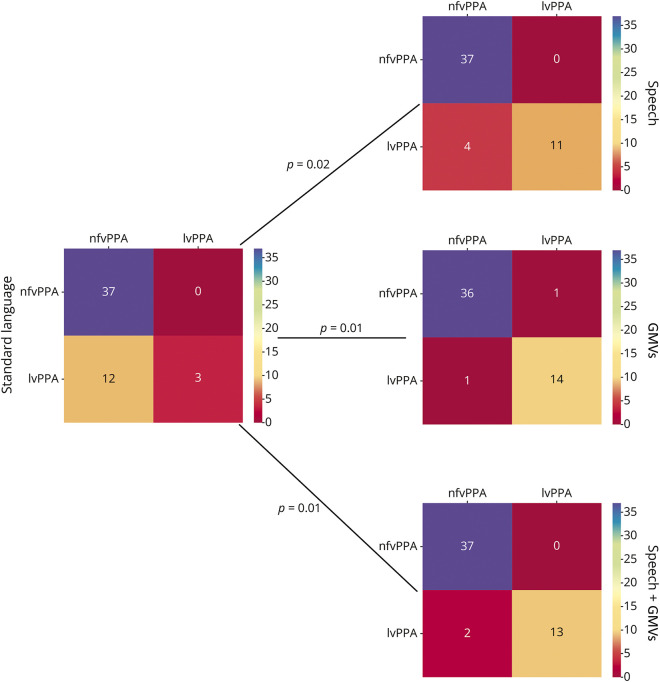
Significant Differences Between Sequential Feature Selection Models for the nfvPPA and lvPPA Classification Values within squares denote the number of patients correctly or not correctly classified by each model. *p* denotes McNemar differences between the model including standard language scores (standard language) and those including “Picnic Scene” (speech) parameters, GMVs, and their combination as independent variables. Color bars denote the number of patients. GMVs = gray matter volumes; lvPPA = logopenic variant of PPA; nfvPPA = nonfluent/agrammatic variant of PPA.

Concerning PPA differentiation, each model distinguished svPPA from each other PPA group with high accuracy (*R*^2^ range: svPPA vs nfvPPA = 0.94–1.00; svPPA vs lvPPA = 0.93–1.00; *p* < 0.001). No differences in accuracy were observed among models for this distinction. In distinguishing nfvPPA from the lvPPA group, the models including speech parameters (*R*^2^ = 0.92; *p* < 0.001), GMVs (*R*^2^ = 0.95; *p* < 0.001), and their combination (speech + GMVs; *R*^2^ = 0.97; *p* < 0.001) showed significant higher accuracies compared with the model including standard language scores only (*R*^2^ = 0.75; *p* = 0.01) (Figure 1). Adding standard language scores to each of the best models (speech, GMVs, and speech + GMVs) did not result in any further improvement in accuracy.

The sequential feature models run with the remaining highly correlated variables showed similar findings in PPA differentiation (*R*^2^ range: svPPA vs nfvPPA = 0.94–0.99; svPPA vs lvPPA = 0.91–1.00; nfvPPA vs lvPPA = 0.87–0.97).

### Longitudinal Findings

#### Sample

A subgroup of 34 patients (10 patients with nfvPPA [age 67 ± 11, 4 women, CDR-FTLD 3 ± 1], 17 with svPPA [age 65 ± 9, 11 women, CDR-FTLD 4 ± 3], and 7 with lvPPA [age 72 ± 8, 5 women, CDR-FTLD 4 ± 2]) underwent a neuropsychological follow-up visit after a mean period of 10.2 months (range 5.50–24.80 months) from the first visit. Sociodemographic and clinical characteristics of the longitudinal sample are summarized in Table 5. The performance of this sample on the “Picnic Scene” test, as detailed in eTable 4, aligns with the findings previously described for the entire sample.

**Table 5:** Baseline Sociodemographic and Clinical Features of Patients Belonging to the Longitudinal Sample

	nfvPPA (N = 10)	svPPA (N = 17)	lvPPA (N = 7)	*p* Value		
				nfvPPA vs svPPA	nfvPPA vs lvPPA	svPPA vs lvPPA
Age (y)	66.77 ± 11.47 (51.57–83.87)	64.89 ± 9.03 (42.06–74.08)	71.84 ± 8.18 (58.68–80.37)	1.00	0.88	0.36
Sex (female/male)	4/6	11/6	5/2	0.26	0.34	1.00
Education (y)	8.80 ± 3.88 (5.00–17.00)	11.53 ± 3.92 (5.00–17.00)	11.00 ± 2.77 (5.00–13.00)	0.22	0.72	1.00
Disease duration (mo)	32.73 ± 21.55 (3.02–68.01)	49.98 ± 29.07 (11.33–126.59)	25.62 ± 20.74 (4.14–51.02)	0.32	1.00	0.17
CDR	0.29 ± 0.27 (0.00–0.50)	0.55 ± 0.28 (0.00–1.00)	0.40 ± 0.22 (0.00–0.50)	0.18	1.00	0.95
CDR-SB	1.86 ± 0.85 (1.00–3.00)	2.20 ± 2.24 (0.50–7.00)	2.25 ± 1.13 (1.00–4.00)	1.00	1.00	1.00
CDR-FTLD	3.42 ± 1.32 (2.00–5.50)	3.94 ± 3.22 (1.00–11.00)	4.00 ± 2.00 (2.00–6.00)	1.00	1.00	1.00
CSF phosphorylated tau/β-amyloid 42, pg/mL^a^	0.08 ± 0.03 (0.04–0.12)	0.07 ± 0.04 (0.04–0.17)	0.23 ± 0.15 (0.07–0.44)	1.00	0.01	0.01
MMSE score	25.30 ± 2.45 (22.00–30.00)	19.94 ± 8.23 (5.00–30.00)	22.86 ± 5.46 (15.00–29.00)	0.14	1.00	0.98
ADL	5.90 ± 0.32 (5.00–6.00)	5.50 ± 1.02 (3.00–6.00)	6.00 ± 0.00 (6.00–6.00)	0.59	1.00	0.52
IADL^b^	90.00 ± 15.37 (62.50–100.00)	71.94 ± 33.47 (0.00–100.00)	100.00 ± 0.00 (100.00–100.00)	0.27	1.00	0.09

Abbreviations: ADL = activities of daily living; ANOVA = analysis of variance; BDI = Beck Depression Inventory; CDR = Clinical Dementia Rating Scale; CDR-FTLD = Clinical Dementia Rating–Frontotemporal Lobar Degeneration; CDR-SB = Clinical Dementia Rating Sum of Boxes; CSF phosphorylated tau/β-amyloid 42 = CSF levels of phosphorylated tau/CSF levels of β-amyloid 42; HC = healthy control; IADL = instrumental activities of daily living; lvPPA = logopenic variant of PPA; MMSE = Mini-Mental State Examination; N = number; nfvPPA = non fluent/agrammatic variant of PPA; PPA = primary progressive aphasia; svPPA = semantic variant of PPA.

Values are frequencies or mean ± SD (range). We considered significant *p* values less than 0.05. *p* Values (*p* < 0.05) refer to ANOVA models followed by post hoc pairwise comparisons (Bonferroni-corrected for multiple comparisons) or the Fisher exact test. Disease duration was defined as months from onset to the date of neuropsychological visit.

a Twenty patients (8 with nfvPPA, 8 with svPPA, and 4 with lvPPA) underwent lumbar puncture.

b Ratio between the score obtained by the patient and the maximum number of administered items.

#### Language and Speech Changes

The results of the test for linear trend performed to assess the longitudinal changes over time in Picnic Scene test parameters and in standard language tests are summarized in Table 6.

**Table 6:** Longitudinal Performances on the Picnic Scene Test and Standard Language Tests of Patients With PPA

	nfvPPA (N = 10)	*p* Value	svPPA (N = 17)	*p* Value	lvPPA (N = 7)	*p* Value	Over time interaction *p* value
Speech rate and speech sound errors							
Total duration (s)	4.95 ± 2.00	0.34	5.53 ± 1.77	1.00	4.83 ± 1.80	0.54	0.35
Duration without pauses (s)	−11.22 ± 4.53	0.87	−12.53 ± 4.01	1.00	−10.94 ± 4.08	0.10	0.14
Total number of words	−13.25 ± 5.36	0.89	−14.8 ± 4.74	1.00	−12.93 ± 4.82	0.12	0.39
WPS	−0.10 ± 0.04	1.00	−0.11 ± 0.03	0.99	−0.09 ± 0.03	0.01	1.00
WPS-np	0.10 ± 0.04	1.00	0.12 ± 0.04	0.36	0.10 ± 0.04	0.41	0.89
Phonotactic distortions	—	—	—	—	—	—	—
AOS	—	—	—	—	—	—	—
PE	3.19 ± 1.29	0.03	3.56 ± 1.14	0.03	3.11 ± 1.16	0.39	0.38
PEW	0.05 ± 0.02	0.07	0.05 ± 0.02	0.048	0.05 ± 0.02	0.31	0.10
Other disruptions to fluency							
False starts	1.77 ± 0.71	0.10	1.97 ± 0.63	0.36	1.72 ± 0.64	1.00	0.32
False start rate	0.01 ± 0.00	0.14	0.01 ± 0.00	0.37	0.01 ± 0.00	1.00	0.81
Filled pauses	4.38 ± 1.77	0.29	4.89 ± 1.57	0.51	4.27 ± 1.59	1.00	1.00
SCS	−0.31 ± 0.12	0.85	−0.34 ± 0.11	1.00	−0.30 ± 0.11	1.00	0.91
SCS rate	0.00 ± 0.00	1.00	0.00 ± 0.00	1.00	0.00 ± 0.00	1.00	1.00
IS	−0.38 ± 0.16	0.12	−0.43 ± 0.14	1.00	−0.37 ± 0.14	0.54	0.77
IS rate	0.00 ± 0.00	0.24	−0.01 ± 0.00	0.77	0.00 ± 0.00	0.37	0.88
Lexical content							
Open class words	−7.22 ± 2.92	1.00	−8.06 ± 2.58	0.83	−7.05 ± 2.62	0.06	0.26
Closed class words	−7.68 ± 3.11	0.82	−8.58 ± 2.75	1.00	−7.50 ± 2.79	0.16	0.56
Verbs	−4.92 ± 1.99	1.00	−5.49 ± 1.76	1.00	−4.80 ± 1.79	0.07	0.68
Nouns	−5.11 ± 2.07	1.00	−5.71 ± 1.83	0.55	−4.98 ± 1.86	0.08	0.21
Noun rate	−0.04 ± 0.02	0.93	−0.04 ± 0.01	0.36	−0.04 ± 0.01	0.004	1.00
rOC	−0.07 ± 0.03	0.06	−0.08 ± 0.03	0.22	−0.07 ± 0.03	0.25	0.39
rCC	0.67 ± 0.27	0.07	0.75 ± 0.24	0.63	0.66 ± 0.24	0.35	1.00
PrV	−0.01 ± 0.00	1.00	−0.01 ± 0.00	1.00	−0.01 ± 0.00	0.61	1.00
Mean frequency	3.46 ± 1.40	0.61	3.87 ± 1.24	1.00	3.38 ± 1.26	1.00	1.00
Log frequency	0.06 ± 0.03	0.50	0.07 ± 0.02	1.00	0.06 ± 0.02	1.00	1.00
SemE	2.04 ± 0.82	0.64	2.27 ± 0.73	0.003	1.99 ± 0.74	1.00	0.07
SemEW	0.02 ± 0.01	1.00	0.03 ± 0.01	0.004	0.02 ± 0.01	0.82	0.09
Syntactic structure and complexity							
Utterances	−3.11 ± 1.26	0.50	−3.47 ± 1.11	0.86	−3.04 ± 1.13	0.33	0.22
UL	0.07 ± 0.03	0.39	0.07 ± 0.02	0.51	0.06 ± 0.02	1.00	0.59
Sentences	−1.27 ± 0.51	1.00	−1.42 ± 0.45	1.00	−1.24 ± 0.46	0.22	0.98
NWS	−18.71 ± 7.56	1.00	−20.89 ± 6.69	0.84	−18.25 ± 6.80	0.03	0.64
SL	−1.11 ± 0.45	0.20	−1.24 ± 0.40	0.11	−1.08 ± 0.40	0.63	1.00
rSentences	0.02 ± 0.01	1.00	0.02 ± 0.01	0.73	0.02 ± 0.01	1.00	0.89
Embeddings	−2.19 ± 0.89	0.87	−2.45 ± 0.78	0.07	−2.14 ± 0.80	0.31	0.63
SES	−0.61 ± 0.25	0.43	−0.69 ± 0.22	1.00	−0.60 ± 0.22	0.34	0.87
SIS	−0.58 ± 0.23	0.72	−0.64 ± 0.21	1.00	−0.56 ± 0.21	0.44	1.00
PrSES	0.01 ± 0.00	1.00	0.01 ± 0.00	1.00	0.01 ± 0.00	1.00	1.00
SP rate	−0.10 ± 0.04	0.22	−0.11 ± 0.04	0.04	−0.10 ± 0.04	0.32	1.00
MsynE	1.77 ± 0.71	0.40	1.97 ± 0.63	0.052	1.72 ± 0.64	1.00	0.47
rMsynE	0.08 ± 0.03	0.89	0.09 ± 0.03	0.02	0.08 ± 0.03	0.54	1.00
Standard language assessment							
CaGi, naming	−4.21 ± 1.84	0.27	−5.08 ± 1.63	0.02	−5.61 ± 1.02	0.61	—
CaGi, comprehension	−1.79 ± 0.78	0.71	−2.16 ± 0.69	0.38	−2.39 ± 0.43	—	—
AAT, repetition	−9.94 ± 4.35	0.24	−11.99 ± 3.84	0.25	−13.25 ± 2.4	0.65	—

Abbreviations: AAT = Aachener Aphasia Test; AOS = apraxia of speech; HC = healthy control; IS = incomplete sentences; log frequency = frequency logarithm; lvPPA = logopenic variant of PPA; MsynE = morphosyntactic errors; N = number; nfvPPA = nonfluent/agrammatic variant of PPA; NWS = number of words in sentences; PE = phonological errors; PEW = phonological errors per word; PPA = primary progressive aphasia; PrSES = proportion of sentences with expressed subject; PrV = proportion of verbs; rCC = closed class word ratio; rMsynE = morphosyntactic error ratio; rOC = open class word ratio; rSentences = sentence ratio; SCS = self-corrected sequences; SemE = semantic errors; SemEW = semantic errors per word; SES = sentences with express subject; SIS = sentences with implied subject; SL = sentence length; SP rate = syntax production rate; svPPA = semantic variant of PPA; UL = utterance length; WPS = words per second; WPS = words per second-no pauses.

Values are predicted delta changes ± SD. We considered significant *p* values less than 0.05. *p* Values (*p* < 0.05) refer to linear trend models followed by post hoc comparisons (Bonferroni-corrected for multiple comparisons).

Concerning speech changes in each variant over time, patients with nfvPPA produced more phonological errors; those with svPPA made more phonological and semantic errors, produced more phonological and semantic errors per word, had a lower syntax production rate, showed higher proportion of morphosyntactic errors, and performed worse in naming; and patients with lvPPA showed reduced WPS, reduced noun rate, and reduced number of words in sentences.

The group-by-time interaction analysis did not show significant differences between PPA groups.

## Discussion

In a well-characterized group of patients with PPA, we identified specific features of connected speech that, either alone or in combination with language scores and GMVs, most effectively distinguished the 3 PPA variants. In addition, we tracked longitudinal changes in speech parameters for each variant over time. We acknowledge that the PPA groups were comparable in disease severity, as assessed by MMSE, CDR Scale, and Frontal Assessment Battery scores.

When analyzing the connected speech, consistent with the current literature, patients with PPA compared with HCs presented a reduced speech rate and more speech sound errors, disruptions of fluency, poor lexical content, and syntactic complexity.^6,8,9,30^ These specific speech characteristics led to a clear distinction between each PPA variant group and HCs, with high accuracy (*R*^2^ = 0.93–0.98). The lowest accuracy (*R*^2^ = 0.93), although still significant, was observed in distinguishing patients with svPPA from HCs. This is likely because those with svPPA share a similar speech pattern with controls, differing mainly in reduced lexical content and a higher prevalence of verbs and pronouns over nouns, as noted in this study and previous research.^8,9,13,14^

When comparing the PPA groups with each other, our study's primary finding is that svPPA were reliably distinguished from the other 2 variants using all models, including that based solely on standard language measures. Specifically, patients with svPPA were accurately identified by their lower naming performance and higher repetition scores, which were sufficient to differentiate them from the other PPA variant groups. This result aligns with previous findings because standard language tests alone have already proven effective in identifying svPPA.^18^

Conversely, when comparing nfvPPA and lvPPA, standard measures alone proved insufficient for achieving satisfactory accuracy (*R*^2^ = 0.75), and incorporating these measures into other models did not enhance their effectiveness. This highlights the well-known difficulty of accurately distinguishing these variants with standard language assessments alone.^17,18^ Specifically, the impairment in verbal repetition, characteristic of patients with lvPPA, can also be seen in those with nfvPPA,^31-36^ primarily due to disruption in articulatory planning or rehearsal of verbal information.^32^ These clinical similarities make it challenging to differentiate nfvPPA from lvPPA, both at onset and throughout the disease course.^18^ By contrast, quantitative speech analysis alone achieved a significant improvement in accuracy, reaching 92%. Specifically, reduced total speech duration, fewer utterances, and a higher use of open class words (e.g., nouns) were the most effective measures for distinguishing nfvPPA from lvPPA. This indicates that a 2-minute recorded speech sample during the neuropsychological screening can provide valuable metrics for accurately identifying these variants. Furthermore, while these findings confirm previously observed speech differences between the 2 groups,^8^ they emphasize that these particular measures are among the most effective for clinical practice.

While the models' overall effectiveness was similar, adding GMV data to speech analysis improved accuracy in distinguishing nfvPPA from lvPPA groups (*R*^2^ = 0.97). The brain regions most indicative for the model were the left supplementary motor area and the left insula, both of which showed greater atrophy in nfvPPA, as well as the left middle temporal gyrus, which was more preserved in this group compared with lvPPA. The atrophy in the left supplementary motor area and left insula is associated with speech errors, grammar programming dysfunction, and a reduced speech rate in nfvPPA^9^ while the damage to the left middle temporal lobe is linked to lexical access difficulties in both lvPPA and svPPA.^8^ Although not identified by the SFS model, patients with lvPPA demonstrated greater atrophy in the left occipital gyrus compared with those with nfvPPA. This finding may suggest a subtle cognitive impairment in lvPPA that extends beyond language functions^37^ and, as reported in the literature, is likely associated with amyloid deposition in occipital regions.^38^

Of interest, some variables identified by SFS models as key for distinguishing PPA variants were not individually significant because SFS evaluates combinations of variables that collectively enhance group differentiation.

Over time, patients with nfvPPA exhibited a significant increase in phonological errors, confirming previous longitudinal studies that reported a notable rise in speech errors in patients with nfvPPA over a comparable time frame.^19^ As we mentioned above, repetition involves complex processes, including the short-term storage of phonological information and articulatory rehearsal, both essential for maintaining and producing verbal information.^39,40^ Failures in one or both of these processes can directly lead to increased phonological errors.^41^ We suspected that both processes might be impaired in patients with nfvPPA.^41^ Phonetic distortions, crucial for distinguishing lvPPA from nfvPPA, are common in English-speaking patients with nfvPPA but rarely observed in Italian because of articulatory differences, complicating diagnosis in Italy.^15^ In addition, distinguishing phonological errors from motor speech errors in Italian is challenging, raising the possibility that the observed longitudinal changes may partly reflect worsening AOS in patients with nfvPPA.

In patients with svPPA over time, we observed an increase in semantic and morphosyntactic errors, reduced syntactic production, and a further decline in naming abilities. These findings align with previous studies that reported similar trends in svPPA, including a progressive decrease in sentence construction performance^42^ and a significant decline in grammatical complexity.^19^ Syntax alterations in svPPA may result from broader involvement of the language system beyond traditionally affected regions. In addition, in our svPPA group, worsening in syntax production could be explained by reduced understanding of the semantic aspects related to the grammatical properties of words, such as their roles or relationships within a sentence.^9^ Conversely, the naming difficulties and semantic errors characteristic of svPPA were clearly predicted by alterations of semantic brain circuit.

Patients with lvPPA exhibited a progressive decline in WPS, reduced noun rate, and reduced number of words per sentence over time. These findings are consistent with a longitudinal study that reports significantly greater impairments in both speech rate and speech error rates in patients with lvPPA over time.^19^ This progression is believed to arise from increasingly impaired single-word retrieval.^19^

This study should be considered in light of its potential limitations. First, despite being reviewed by 2 independent evaluators and a supervisor, the speech analysis remains vulnerable to operator-dependent biases due to the complexity of the variables and inherent subjectivity in the evaluation. Second, we performed a cross-validation analysis and we acknowledge the lack of an external validation cohort. However, our goal was not to surpass current methods but to identify features aiding diagnosis in challenging or early-stage cases, with future validation needed in uncertain or real-world scenarios. In fact, an important goal of this proof-of-concept study was to determine which among the many variables typically collected during the diagnostic process are most predictive of the final diagnosis. From a clinical perspective, this is a crucial finding because it has the potential to streamline diagnostic workflows, reduce the time spent on unnecessary evaluations, and optimize the assessment process by focusing on the most informative measures. Third, the small sample size for the longitudinal analysis may limit the generalizability of our findings. Fourth, we used 2 separate groups of HCs, one for MRI and another for speech analysis. Finally, word-finding difficulties (anomie) were not addressed in the speech analyses because of challenges in distinguishing between problems related to lexical retrieval and semantic access.

In conclusion, this study analyzes a large cohort of patients with PPA both cross-sectionally and longitudinally, integrating connected speech, standardized language tests, and GMVs. Although standard language measures alone were sufficient to identify the svPPA group, they struggled to distinguish nfvPPA from lvPPA, where connected speech analysis significantly improved diagnostic accuracy. In this study, the incorporation of quantitative speech metrics, such as reduced total speech duration and fewer utterances, provides an accessible method for improving diagnostic precision, even with brief speech samples collected during routine neuropsychological evaluations. In addition, combining GMV data further enhanced differentiation, highlighting the value of multimodal approaches. The identification of key brain regions, such as the left supplementary motor area and the left insula in nfvPPA, supports the integration of GMV analysis into clinical workflows. Longitudinally, we identified features of speech decline, such as increasing phonological errors in nfvPPA, declining semantic and syntactic abilities in svPPA, and reduced WPS in lvPPA, and provided valuable insights into disease trajectories and patients' prognosis and for planning speech language therapy and pharmacologic interventions.

## Glossary

ANCOVA: analysis of covariance
ANOVA: analysis of variance
AOS: apraxia of speech
BDI: Beck Depression Inventory
CDR: Clinical Dementia Rating
FTLD: Frontotemporal Lobar Degeneration
GM: gray matter
GMVs: gray matter volumes
HC: healthy control
MMSE: Mini-Mental State Examination
PPA: primary progressive aphasia
lvPPA: logopenic variant PPA
nfvPPA: nonfluent variant PPA
svPPA: semantic variant PPA
SFS: sequential feature selection
WAB: Western Aphasia Battery
WPS: words per second
WPS-np: words per second-no pauses
